# High Starch Induces Hematological Variations, Metabolic Changes, Oxidative Stress, Inflammatory Responses, and Histopathological Lesions in Largemouth Bass (*Micropterus salmoides*)

**DOI:** 10.3390/metabo14040236

**Published:** 2024-04-19

**Authors:** Yuanyuan Xie, Xianping Shao, Penghui Zhang, Hao Zhang, Jiaxing Yu, Xinfeng Yao, Yifan Fu, Jiao Wei, Chenglong Wu

**Affiliations:** National-Local Joint Engineering Laboratory of Aquatic Animal Genetic Breeding and Nutrition (Zhejiang), Department of Fisheries, School of Life Science, Huzhou University, 759 East 2nd Road, Huzhou 313000, China; xieyuanyuan23@outlook.com (Y.X.); penghuizhang2021@outlook.com (P.Z.); zhlyyxxf@outlook.com (H.Z.); yujiaxing313@outlook.com (J.Y.); xinfengyao666@outlook.com (X.Y.); fuyifan1210@outlook.com (Y.F.); weijiao0323@outlook.com (J.W.)

**Keywords:** *Micropterus salmoides*, high starch, hematological variations, metabolic changes, inflammatory responses

## Abstract

This study evaluated effects of high starch (20%) on hematological variations, glucose and lipid metabolism, antioxidant ability, inflammatory responses, and histopathological lesions in largemouth bass. Results showed hepatic crude lipid and triacylglycerol (TAG) contents were notably increased in fish fed high starch. High starch could increase counts of neutrophils, lymphocytes, monocytes, eosinophils, and basophils and serum contents of TAG, TBA, BUN, and LEP (*p* < 0.05). There were increasing trends in levels of GLUT2, glycolysis, gluconeogenesis, and LDH in fish fed high starch through the AKT/PI3K signal pathway. Meanwhile, high starch not only triggered TAG and cholesterol synthesis, but mediated cholesterol accumulation by reducing *ABCG5*, *ABCG8*, and *NPC1L1*. Significant increases in lipid droplets and vacuolization were also shown in hepatocytes of D3–D7 groups fed high starch. In addition, high starch could decrease levels of mitochondrial *Trx2*, *TrxR2*, and *Prx3*, while increasing ROS contents. Moreover, high starch could notably increase amounts of inflammatory factors (IL-1β, TNF-α, etc.) by activating NLRP3 inflammasome key molecules (*GSDME*, *caspase 1*, etc.). In conclusion, high starch could not only induce metabolic disorders via gluconeogenesis and accumulation of glycogen, TAG, and cholesterol, but could disturb redox homeostasis and cause inflammatory responses by activating the NLRP3 inflammasome in largemouth bass.

## 1. Introduction

As the cheapest and widest energy source or ingredient, starch is also a good binder for animal feeds [1,2]. In most cases, adequate amount of dietary starch could enhance growth and feed efficiency, but it can also save protein by redirecting amino acids away from the oxidative process in humans, terrestrial animals, and fish species [3,4]. Meanwhile, optimal contents of dietary starch could improve metabolism and enhance antioxidant capacity and immunity in animals [5,6,7]. Nevertheless, carnivorous fish exhibit reduced capacity to digest dietary starch compared to omnivorous and herbivorous fish, as indicated by various research studies involving grass carp (*Ctenopharyngodon idellus*), Chinese longsnout catfish (*Leiocassis longirostris*) [8], jundiá catfish (*Rhamdia quelen*), Nile tilapia (*Oreochromis niloticus*) [9], and largemouth bass (*Micropterus salmoides*) [10]. Several studies have proved that long-term intake of excessive dietary starch could induce metabolic dysfunction [6,11] and then impair the innate immunity in fish [12,13]. Therefore, the regulatory mechanisms of high starch have been a hot research area in carnivorous fish species and other animals.

Too much starch in the diet can cause an increase in serum glucose (GLU) levels, impacting glucose metabolism and lipid metabolic indices, like high-density lipoprotein cholesterol (HDL-C), triacylglycerol (TAG), total cholesterol (TC), low-density lipoprotein cholesterol (LDL-C), etc. [1,7,12]. Previous studies have found glucose and lipid metabolic processes can be regulated by different signaling pathways mediated by serum hormones, including glucagon (GC), insulin (INS), adiponectin (ADPN), and leptin (LEP) [7,14,15]. In addition, the processes of glycogen synthesis and decomposition are also modulated by these serum hormones in animals in response to higher dietary starch [14,16,17]. Moreover, several studies have reported that higher dietary starch could induce hepatic cholesterol and bile acid accumulation in humans and animals [18]. These metabolic processes, such as glycolysis and gluconeogenesis, lipolysis and lipogenesis, glycogen synthesis, and cholesterol and bile acid accumulation, are mainly regulated by various signaling pathways, including AKT and SREBP signaling pathways in humans and animals [19,20]. While most research has concentrated on glycolysis, gluconeogenesis, lipolysis, and glycogen synthesis in carnivorous fish, there are limited data available regarding the synthesis and storage of bile acids through hepatic cholesterol in carnivorous fish species consuming higher dietary starch.

Throughout the breakdown of nutrients, such as starch, reactive oxygen species (ROS) were consistently formed in humans, terrestrial animals, and fish species [3,21]. Although optimal amounts of ROS could play essential functions in growth, metabolism, and immune defense, ROS overload could induce the damage of proteins, lipids, and DNA [22,23]. So, it is important to alleviate these oxidative stresses induced by ROS overload through different adequate antioxidant enzymes and molecules, including superoxide dismutase (SOD), catalase (CAT), thioredoxin 2 (*Trx2*), thioredoxin reductase 2 (*TrxR2*), peroxiredoxin 3 (*Prx3*), and glutathione S-transferase (GST) [13,24,25]. Past research has indicated that carnivorous fish fed diets high in starch exhibited low antioxidant capacity and strong oxidative stress [13,23,26]. In addition, excessive ROS can cause cellular damage in these immune regulatory cells, including leukocytes, macrophages, and natural killer cells [27]. Prasad and colleagues [21] have shown that immune and inflammatory responses can be influenced by the release of various cytokines, such as tumor necrosis factor-α (TNF-α), interferons (IFNs), and interleukins (ILs), which was also supported by Herb and Schramm [28] and Liu et al. [29]. In animals, the leukocyte numbers and platelet (PLT) counts have appeared as biomarkers of inflammation or inflammatory response in clinical practices recently [3,30,31,32]. And the secretion and maturation of these pro-inflammatory cytokines could be mediated by the NLRP3 inflammasome in animal cells [33]. Excessive production of ROS can activate the NLRP3 inflammasome [34,35,36]. Yet, there were limited data regarding the connection among elevated starch-induced ROS, NLRP3 inflammasome, and inflammatory markers in carnivorous fish.

Largemouth bass is a common fish species that is economically important and extensively cultivated in China due to its rapid growth, abundant harvest, soft flesh, lack of intermuscular spines, and other favorable characteristics [37]. Numerous previous research projects have investigated the prolonged impacts of increased dietary starch on the development characteristics, metabolic capacities, and overall health of the liver and intestines in largemouth bass [17,18,38]. Little information could be obtained on the short-time relationship between high starch-mediated hematological variations, metabolic changes, oxidative stress, inflammatory responses, and histopathology in largemouth bass. This research sought to investigate the processes that lead to the development of metabolic fatty liver in largemouth bass fed a diet high in starch. This study utilized a system dynamics approach, analyzing data from various body and blood measurements as well as metabolic, oxidative stress, antioxidant, inflammatory, and histopathological markers in largemouth bass fed with high starch.

## 2. Materials and Methods

### 2.1. Diets, Fish, and Feeding Trials

Two isonitrogenous and isolipidic (crude protein: 46.72%, crude lipid: 9.86%) trial diets were produced with two levels of dietary starch (0% and 20%), respectively (Table 1). All used ingredients were firstly filtrated with 60-mesh screens, mixed, produced into pellets with 2.5 mm diameter, air-dried, and then stored following methods outlined by Wu et al. [39]. Juvenile largemouth bass were acquired from a nearby fish breeder and introduced to recirculating tanks (1000 L) with a starch-free diet for two weeks. Following the acclimation period, 720 healthy fish (with an average starting weight of 7.43 ± 0.15 g) were chosen and distributed at random among 24 recirculating tanks (each with a capacity of 500 L and containing 30 fish). After the distribution, all fish were still fed a diet without starch for one month to maintain physiological consistency. Then, control groups with 3 tanks were still fed with a control diet without starch; these experimental groups (21 tanks) were fed with a high-starch diet (20%) and randomly distributed into seven groups (D1–D7) for 7 days. During the culturing time, all fish were fed with 3% of the total fish weight per day at 08:00 and 17:00. The recirculated water was filtered, with a temperature range of 26.3 to 28.4 degrees Celsius, pH range of 6.8 to 7.1, dissolved oxygen levels above 5.8 mg/L, low nitrite levels less than 0.02 mg/L, low ammonia nitrogen levels less than 0.05 mg/L, and exposed to natural sunlight.

### 2.2. Fish Tissue Samples’ Preparation

After the experiment, all fish in these trial groups were first fasted for about 24 h, anesthetized with MS-222 (Sigma, St. Louis, MO, USA), and weighed to evaluate fish body indices. Twenty fish in each tank were randomly selected, weighed, and used for blood sampling for hematological assays. All the serum and liver samples were gathered with methods supplied by Wu et al. [39]. These tested samples were firstly frozen using liquid nitrogen and subsequently kept at −80 °C for additional measurements. Frozen liver tissue samples were powderized in liquid nitrogen, suspended with physiological saline, and centrifuged with methods supplied by Wu et al. [39]. All the supernatants were gathered, divided into Eppendorf tubes, and then frozen at −80 °C for further experimental tests.

### 2.3. Measurement of Hematological and Serum Biochemical Indices

The TEK 8500 VET automated blood analyzer from Jiangxi Tekang Tech (Nanchang, China) was utilized for the assessment of hematological parameters and cell populations, such as red blood cells (RBCs), hemoglobin (HGB), white blood cells (WBCs), eosinophils (EOS), neutrophils (NEU), basophils (BAS), lymphocytes (LYM), monocytes (MON), and platelets (PLT). All measurements were performed with 15 replicates.

Serum levels or activities of various biomarkers including GLU, INS, HDL-C, LEP, TAG, LDL-C, TC, aspartate aminotransferase (AST), total bile acid (TBA), albumin (ALB), alanine aminotransferase (ALT), alkaline phosphatase (ALP), blood urea nitrogen (BUN), LDH, GC, and ADPN were measured using commercial diagnostic kits provided by Jiancheng Biotech (Nanjing, China). All analyses were performed with 9 replicates.

### 2.4. Measurement of Glucose and Lipid Metabolic Parameters in Fish Liver

Diagnostic kits from Jiancheng Biotech (Nanjing, China) were used to measure the activities or contents of various enzymes and compounds including glucokinase (GCK), phosphofructokinase (PFK), pyruvic acid (PA), pyruvate kinase (PK), lactic dehydrogenase (LDH), phosphoenolpyruvate carboxylase (PEPC), lactic acid (LA), fructose-1.6-bisphosphatase (FBP), liver triglyceride (LTAG), acetyl coenzyme A (Ac-CoA), malonyl monoacyl CoA (Mal-CoA), and liver glycogen (LAG). Levels of glucose transporter 2 (GLUT2), glycogen synthetase (GCS), glucose 6-phosphatase (G6Pase), glycogen branching enzyme (GBE), fatty acid synthase (FAS), glycogen debranching enzyme (GDE), and acetyl-CoA carboxylase (ACC) were assessed through relative ELISA kits following the manufacturer’s instructions (Hengyuan Biotech, Shanghai, China). The crude lipid content in the liver was measured using the chloroform-methanol method supplied by Araujo et al. [40]. All measurements were performed with 9 replicates.

### 2.5. Measurement of Antioxidant and Inflammatory Parameters in the Liver

Commercial diagnostic kits from Jiancheng Biotech (Nanjing, China) were used to measure levels of total superoxide dismutase (T-SOD), glutathione S-transferase (GST), CAT, malondialdehyde (MDA), hydrogen peroxide (H_2_O_2_), and total antioxidant capacity (T-AOC). ELISA kits from Jiangsu Meimian Industrial Co., Ltd. (Hangzhou, China) were used to evaluate the contents of ROS in the liver. Levels of TNF-α, IL-1β, IL-6, IFN-γ, IL-8, IL-12, and IL-17 in these fish liver samples were also assessed with ELISA kits purchased from Shanghai Hengyuan Biotech (Shanghai, China) following their recommended protocols. All measurements were performed with 9 replicates.

### 2.6. Measurement of Gene Expression in the Liver

RNA extraction, RNA integrity examination, quantification, and RNA reverse transcription were performed with methods supplied by Wu et al. [13]. All the cDNA samples were treated with ethanol (95%) and then frozen at −80 °C for further analysis. All the primers were designed according to relative gene sequences from largemouth bass in the GenBank (Table 2). The CFX96 machine from Bio-Rad in Hercules, CA, USA was utilized for conducting real-time qPCR analysis, with expression variations analyzed using the 2^−ΔΔCT^ method as outlined by Yang et al. [37]. All measurements were performed with 9 replicates for further analyses.

### 2.7. Measurement of Histomorphometrical Parameters of the Liver

Six fish were selected at random from each category, and liver tissue samples measuring 0.5 cm × 0.5 cm × 0.5 cm were rinsed with 0.6% saline and then immersed in a 4% paraformaldehyde solution for 48 h. The liver tissue samples were then fixed, dehydrated, embedded, and stained with hematoxylin and eosin (HE), periodic acid Schiff (PAS), or oil red O (ORO) before being examined under light microscopy (DM500, Leica, Leica Microsystems (Schweiz) AG, Heerbrugg, Switzerland). Liver micrographs were captured at 20 × and 40 × magnification and recorded using a digital camera following the procedures outlined in our laboratory’s previous publication [39]. K-Viewer (https://kv.kintoneapp.com/en/user/, accessed 31 March 2023) 1.0 software (1.0.4) (Konfoong Bioinf Tech Co., Ltd, Ningbo, China) and Slide Viewer (accessed 21 April 2023) 2.5 software (2.5.0) (3DHISTECH Ltd., Budapest, Hungary) were utilized for image analysis conducted by Wu et al. [39]. Each analysis was conducted with 3 duplicates.

### 2.8. Data Analysis

All results were expressed as mean ± SD (standard deviation). Groups were compared using SPSS 25.0 (IBM, Chicago, IL, USA) through a one-way analysis of variance (ANOVA). Tukey’s multiple interval test was used for multiple comparisons between different dietary treatments. Furthermore, orthogonal polynomial contrasts about linear and/or quadratic effects were used with the methods supplied by Wu et al. [39]. A significant difference was indicated when *p* was less than 0.05.

## 3. Results

### 3.1. Body Indices and Lipid Contents in the Liver

**Figure 1:**
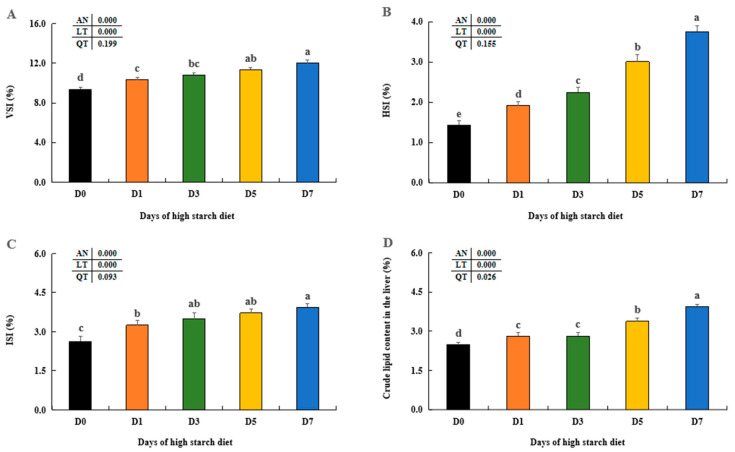
Effect of high-starch diet on body index including VSI (**A**), HSI (**B**), ISI (**C**) and crude lipid content in the liver (**D**) in largemouth bass. Data are reported as the mean ± SD of three replicates (*n* = 3). Bars with different superscripts (^a–e^) are significantly different (*p* < 0.05). To calculate animal-specific parameters, the following equations were used: hepatosomatic index (HSI, %) = liver weight/final body weight (FBW) × 100. Viscerosomatic index (VSI, %) = viscera weight/FBW × 100. Intestinalsomatic index (ISI, %) = intestinal fat weight/FBW × 100.

The viscerosomatic index (VSI), hepatosomatic index (HSI), and intestinal somatic index (ISI) of the fish showed a tendency to rise as the number of days on a high starch diet increased in largemouth bass juveniles, reaching their peak in the D7 trial groups (*p* < 0.05) (Figure 1A–C). Furthermore, no significant variances were seen in ISI among the D3, D5 and D7 trial groups (*p* > 0.05). The liver’s crude lipid contents increased as the high starch diet was cultured for more days, reaching its peak in the D7 groups (*p* < 0.05), although there were no notable differences between the D1 and D3 groups (*p* > 0.05).

### 3.2. Hematological Parameters

With the increase in the consumption of a high-starch diet by largemouth bass over several days, there was first a rise and then a decrease in the trends on RBC counts in the D1–D7 groups (*p* > 0.05), while there were no notable differences in RBC counts between the D0 and D7 groups (*p* > 0.05). Furthermore, HGB levels rose in the D1, D3, and D5 groups and reached their peak in the D5 trial groups before declining in the D7 treated groups (*p* < 0.05). Additionally, there was a notable increase in the counts of WBC and LYM (*p* < 0.05), with WBC peaking in the D7 groups (*p* > 0.05), and LYM peaking in the D5 groups (*p* > 0.05). In the meantime, all NEU, MON, and EOS levels showed marked increases when compared to the D0 group, peaking in the D7 groups (*p* < 0.05). There were no notable differences in MON and EOS counts between the D3 and D5 groups (*p* > 0.05). Conversely, there was a marked inverse correlation between PLT counts and the duration of consuming a high-starch diet in comparison to the D0 groups (*p* < 0.05). However, no significant differences were presented between the D5 and D7 groups (*p* > 0.05) (Table 3).

### 3.3. Biochemical and Hormone Indicators in the Serum

High dietary starch significantly increased contents or levels of GLU, HDL-C, and LDL-C in the serum of D1–D7 groups when compared with that in the D0 groups fed with a no-starch diet (*p* < 0.05). The peaked levels of HDL-C, and LDL-C were all shown in the D7 groups (*p* < 0.05), with no notable differences among D1, D3, D5, and D7 groups (*p* > 0.05). TG and TC contents were notably heightened and peaked in the D5 and D7 groups (*p* < 0.05), respectively. AST activities were firstly heightened and then maintained stable trends in D1, D3, D5, and D7 groups (*p* > 0.05). Similarly, ALT activities and TBA contents were also markedly heightened to the maximal levels in the D5 and D3 groups, respectively, with no significant differences between the D3 and D5 groups (*p* > 0.05). In addition, amounts of ALP, ALB, LDH, and BUN were all first increased in the D1 and D3 groups when compared with that in the D0 groups (*p* < 0.05) and then reduced in the D5 and/or D7 groups. However, amounts of ALP, ALB, LDH, and BUN in the D7 groups were still significantly higher than those in the D0 groups (*p* < 0.05). In addition, the contents of INS and ADPN first rose to their peaks in the D3 groups when compared to those in the D0 groups (*p* < 0.05) and then decreased to the lower levels in the D5 and D7 groups when compared to that in the D3 groups (*p* < 0.05). However, GC amounts were increased and peaked in the D7 groups when compared to that in the D0 groups. The levels of LEP were also firstly heightened and reached maximal values in the D5 groups and then decreased in the D7 groups, although LEP levels in the D7 groups were markedly higher than those in the D0 groups (*p* < 0.05) (Table 4).

### 3.4. Activities or Contents of Glucose and Lipid Metabolism in Fish Liver

Compared to the D0 groups, amounts and activities of GLUT2, GCK, PFK, PK, LDH, PEPC, and ACC were all notably heightened in the livers of D3, D5, and D7 groups fed with high-starch diet (*p* < 0.05) and reached maximal levels in D5 or D7 groups, respectively. The contents of PA and LA were also notably heightened in the livers of D1–D7 groups fed with a high-starch diet (*p* < 0.05), although decreased in the D7 groups when compared with their peak in D5 and D3 groups (*p* < 0.05), respectively. In addition, activities of FBP, G6Pase, GCS, GBE, and FAS were first increased in D3 or D5 treated groups (*p* < 0.05) and then decreased in D7 groups. However, activities of GDE were significantly reduced in the D1–D7 groups when compared with D0 groups (*p* < 0.05), although there were no differences between the D1–D3, and the D5–D7 groups. While, LAG contents were increased in D1–D7 groups and reached the maximal level in the D7 groups (*p* < 0.05). Although there was no significant variation among the D0, D1, and D3 groups (*p* > 0.05), Ac-CoA contents were increased in D5 and D7 groups (*p* < 0.05). Moreover, there were similar variable patterns in the contents of Mal-CoA and LTAG. Compared to the D0 groups, the maximal contents of Mal-CoA and LTAG were both shown in the D5 groups, although no significant variation could be observed between the D5 and D7 groups (*p* > 0.05) (Table 5).

### 3.5. Gene Expression Variations of Glucose Metabolism in Fish Liver

**Figure 2:**
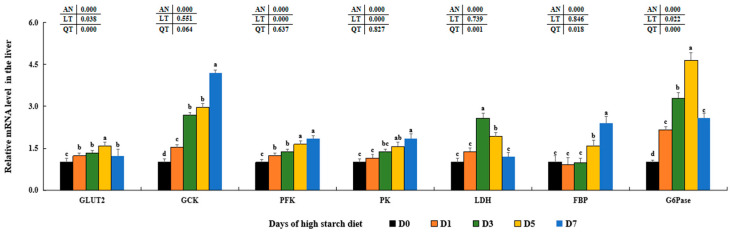
Effect of high-starch diet on related mRNA levels of hepatic glucose metabolism in largemouth bass. Data are reported as the mean ± SD of three replicates (*n* = 3). Bars with different superscripts (^a–d^) are significantly different (*p* < 0.05).

**Figure 3:**
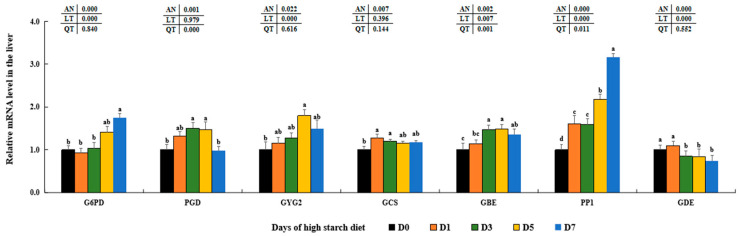
Effect of high-starch diet on related mRNA levels of hepatic glycogen metabolism in largemouth bass. Data are reported as the mean ± SD of three replicates (*n* = 3). Bars with different superscripts (^a–d^) are significantly different (*p* < 0.05).

The levels of *GLUT2*, *GCK*, *PFK*, *PK*, *FBP*, and *G6Pase* showed a rising trend, peaking in the D5 or D7 groups when compared to the D0 groups (*p* < 0.05). In the D3 and D5 groups, the levels of *LDH* transcription showed a notable increase (*p* < 0.05), while the remaining three groups did not exhibit any significant differences statistically (*p* > 0.05) (Figure 2). High dietary starch significantly increased the mRNA levels of glucose-6-phosphate 1-dehydrogenase (*G6PD*), 6-phosphogluconate dehydrogenase (*PGD*), glycgenin 2 (*GYG2*), glycogen synthetase (*GCS*), 1,4-alpha-glucan branching enzyme (*GBE*), and serine/threonine-protein phosphatase PP1 (*PP1*) (*p* < 0.05). The mRNA expression of glycogen debranching enzyme (*GDE*) was significantly reduced in the D5 and D7 groups, with no significant difference between the two groups (*p* > 0.05) (Figure 3).

**Figure 4:**
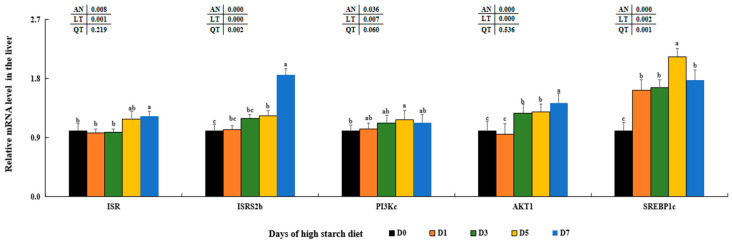
Effect of high-starch diet on related mRNA levels of signal molecules regulating glucose metabolism in largemouth bass. Data are reported as the mean ± SD of three replicates (*n* = 3). Bars with different superscripts (^a–c^) are significantly different (*p* < 0.05).

Furthermore, there were rising trends in the levels of insulin receptor (*ISR*), insulin receptor substrate 2 (*ISR2b*), serine/threonine kinase 1 (*AKT1*), and sterol regulatory element binding protein 1c (*SREBP1c*) observed in the livers of subjects on high-starch diets (*p* < 0.05). In addition, the levels of *PIK3c* were significantly higher in the D5 groups compared to the D0 groups (*p* < 0.05), with no statistical differences observed among the latter (*p* < 0.05). The highest levels of *SREBP1c* were shown in the D5 groups compared to those in the other four groups, although there were no statistical differences observed among D1, D3, and D7 groups (*p* > 0.05) (Figure 4).

### 3.6. Gene Expression Variations of FA and TAG Metabolism in Fish Liver

**Figure 5:**
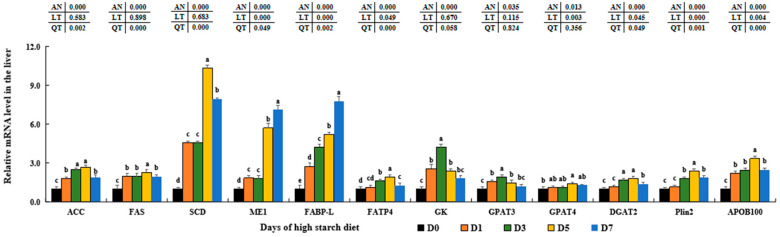
Effect of high-starch diet on related mRNA levels of hepatic lipid metabolism in largemouth bass. Data are reported as the mean ± SD of three replicates (*n* = 3). Bars with different superscripts (^a–e^) are significantly different (*p* < 0.05).

The high-starch diet significantly upregulated the expression of genes involved in fatty acid synthesis, including acetyl-CoA carboxylase (*ACC*), fatty acid synthase (*FAS*), acyl-CoA desaturase (*SCD*), and NADP-dependent malic enzyme 1 (*ME1*), with the highest levels observed in the D5 groups compared to those in D0 groups (*p* < 0.05). Levels of mRNA for fatty acid-binding protein liver type (*FABP-L*) and fatty acid transport protein 4 (*FATP4*) were significantly increased to their highest levels in D7 or D5 groups (*p* < 0.05), respectively. Additionally, high starch was found to notably enhance the expression of glycerol kinase (*GK*), glycerol-3-phosphate acyltransferase 3 (*GPAT3*), *GPAT4*, and diacylglycerol O-acyltransferase 2 (*DGAT2*) related to triglyceride production in the livers of fish fed with high-starch diet (*p* < 0.05). Similarly, the levels of perilipin-2 (*Plin2*) were notably increased to the highest point in the D5 groups and then decreased in the D7 groups (*p* < 0.05), with no significant variations between the D3 and D7 groups (*p* > 0.05). Moreover, the levels of the triglyceride transporter apolipoprotein B-100 (*APOB100*) were significantly increased to the highest point in the D5 groups, then decreased in the D7 groups (*p* < 0.05), with no significant difference between the D1 and D3 groups (*p* > 0.05) (Figure 5).

### 3.7. Cholesterol Metabolism and Bile Acid Synthesis of Gene Expression

**Figure 6:**
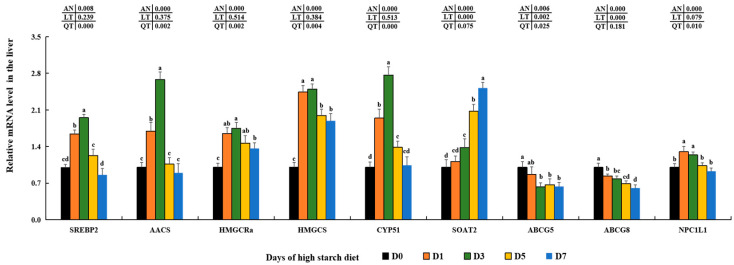
Effect of high-starch diet on related mRNA levels of hepatic cholesterol metabolism in largemouth bass. Data are reported as the mean ± SD of three replicates (*n* = 3). Bars with different superscripts (^a–d^) are significantly different (*p* < 0.05).

In the hepatic cells of fish fed a high-starch diet, the expression levels of genes involved in cholesterol metabolism, including sterol regulatory element-binding protein 2 (*SREBP2*), acetoacetyl-CoA synthetase (*AACS*), 3-hydroxy-3-methylglutaryl-Coenzyme A reductase a (*HMGCRa*), hydroxymethylglutaryl-CoA synthase (*HMGCS*), and lanosterol 14-alpha demethylase (*CYP51*), were significantly elevated in the D3 treated groups compared to the D0 control groups (*p* > 0.05) and then decreased in the D7 groups compared to the D3 treated groups (*p* < 0.05). Both *HMGCRa* and *HMGCS* showed no significant differences not just between the D1 and D3 groups, but also between the D5 and D7 groups (*p* > 0.05). *SREBP2* and *AACS* were not significantly different between D0, D5, and D7 groups (*p* > 0.05), and *CYP51* was not significantly different between D7 and D0 groups (*p* > 0.05). Expression levels of sterol O-acyltransferase 2 (*SOAT2*) consistently rose in high-starch groups as feeding time increased, peaking in the D7 groups (*p* < 0.05). Conversely, the expression of ATP-binding cassette sub-family G member 5 (*ABCG5*) and *ABCG8* were reduced in the D3, D5, and D7 treated groups compared with the D0 control groups (*p* < 0.05). Additionally, there was first an increase trend on the levels of Niemann-Pick C1-like protein 1 (*NPC1L1*) in the D1 and D3 groups and then, they reduced to the original levels in D5 and D7 groups when compared with the D0 groups (Figure 6).

**Figure 7:**
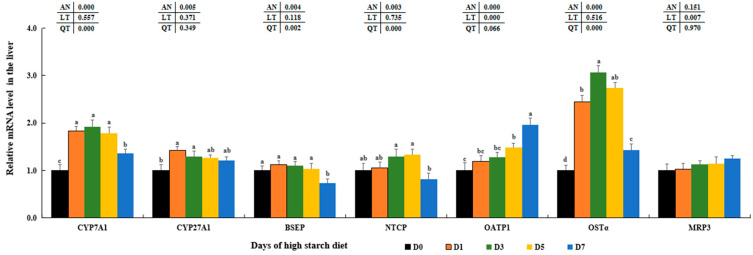
Effect of high-starch diet on related mRNA levels of hepatic bile acid metabolism in largemouth bass. Data are reported as the mean ± SD of three replicates (*n* = 3). Bars with different superscripts (^a–d^) are significantly different (*p* < 0.05).

In addition, the mRNA transcription levels of bile acid processing, such as *CYP7A1* and *CYP27A1*, were significantly increased in the D1 and D3 groups (*p* < 0.05) and then reduced in the D7 groups in comparison to the D0 groups. The expression of the bile salt export pump (*BSEP*) only decreased significantly in the D7 groups compared to D0 (*p* < 0.05), with no significant differences observed among D0, D1, D3, and D5 groups. The transcription levels of sodium/taurocholate cotransporting polypeptide (*NTCP*) were significantly increased in the D3 and D5 treated groups (*p* < 0.05) and then reduced in the D7 treated groups in comparison to the D0 groups. Amounts of solute carrier organic anion transporter family member 1 (*OATP1*) were constantly up-regulated and reached the peak in the D7 treated groups (*p* < 0.05). Moreover, organic solute transporter subunit alpha (*OST-α*) was notably up-regulated in D1–D7 groups and obtained the maximal levels in D3 treated groups compared with D0 control groups (*p* < 0.05). However, no significant variations were observed in the transcription of canalicular multispecific organic anion transporter 2 (*MRP3*) (*p* > 0.05) (Figure 7).

### 3.8. Variation of Antioxidant and Oxidant Parameters

The liver’s T-SOD and CAT activities were notably reduced in D1–D7 groups fed with the high-starch diet compared to the D0 groups (*p* < 0.05). There were no significant differences in T-SOD levels among the D1, D3, and D5 groups (*p* > 0.05), nor in CAT levels between the D1 and D3 groups and among the D3, D5, and D7 groups (*p* > 0.05). ROS and H_2_O_2_ levels were both elevated and peaked in the D5 groups (*p* < 0.05), with no significant variation observed between the D3 and D7 groups in terms of ROS (*p* > 0.05). Amounts of GST and MDA were peaked in the D7 groups (*p* < 0.05), with no significant differences observed among D0, D1, and D3 for GST (*p* > 0.05) and among D1, D3, and D5 treated groups for MDA levels (*p* > 0.05). T-AOC levels were lower in the D5 and D7 groups compared to the D0 groups (*p* > 0.05), with no significant differences observed among the D0, D1, and D3 groups, as well as between the D5 and D7 groups (*p* > 0.05) (Table 6).

**Figure 8:**
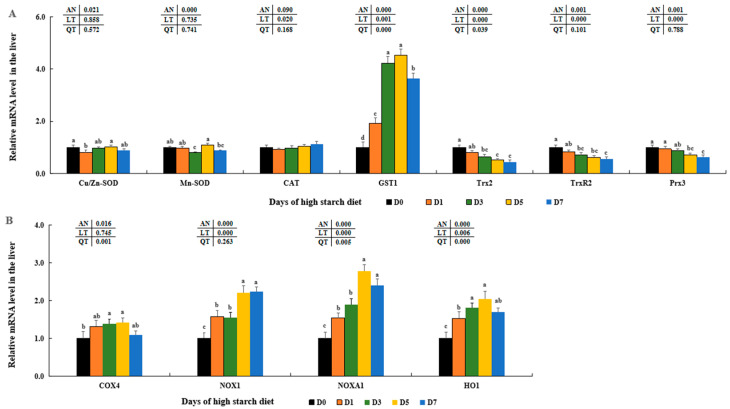
Effect of high-starch diet on related mRNA levels of hepatic antioxidant index in largemouth bass. (**A**) Antioxidant and oxidative parameters, (**B**) ROS generating and oxidative stress indexes. Data are reported as the mean ± SD of three replicates (*n* = 3). Bars with different superscripts (^a–d^) are significantly different (*p* < 0.05).

The expressions of Cu/Zn superoxide dismutase (*Cu/Zn-SOD*) and manganese superoxide dismutase (*Mn-SOD*) were initially reduced to a minimum in the D1 and D3 groups compared to the D0 control groups (*p* < 0.05), respectively, both followed by an increase in the D5 groups (*p* < 0.05) and then were reduced in the D7 groups. There were no significant differences in the transcription levels of *CAT* in the D0–D7 groups (*p* > 0.05). Furthermore, *GST1* transcriptions were notably elevated in the D3 and D5 groups compared to the D0 group, reaching its peak in the D3 and D5 groups (*p* < 0.05), with no significant differences between these two groups (*p* > 0.05). In contrast, the expressions of thioredoxin 2 (*Trx2*), thioredoxin reductase 2 (*TrxR2*), and peroxiredoxin 3 (*Prx3*) were decreased as high-starch feeding increased compared to the D0 groups (*p* < 0.05) (Figure 8A). The livers of largemouth bass fed a high-starch diet showed increased transcription variations of cytochrome c oxidase 4 (*COX4*), NADPH oxidase 1 (*NOX1*), NADPH oxidase activator 1 (*NOXA1*), and heme oxygenase 1 (*HO1*), reaching their peaks in the D5 and/or D7 groups compared to those in the D0 groups (*p* < 0.05). Furthermore, there were no significant variations in *COX4* levels among D0, D1, and D7 groups. There were no notable variations in *HO1* transcription levels between the D5 and D7 treated groups (*p* > 0.05) (Figure 8B).

### 3.9. Variation of Molecules in Inflammatory Responses in Fish Liver

In the livers of largemouth bass fed high starch, TNF-α contents were constantly heightened and peaked in the D7 treated groups compared to the D0 control groups (*p* < 0.05). There were similar variation trends in the levels of IL-6, IL-8, IL-17, and IL-1β in the D1–D7 groups and were notably higher than those in the D0 control groups (*p* < 0.05). IL-6 contents were firstly heightened and peaked in the D3 treated groups compared to the D0 groups (*p* < 0.05). Similarly, contents of IL-8, IL-17, and IL-1β were also firstly heightened and all reached the maximum in the D5 treated groups compared to those in the D0 groups (*p* < 0.05). Contents of IL-12 in high-starch groups were notably higher than that in the D0 groups, although there were no differences among the D1–D7 treated groups (*p* > 0.05). IFN-γ levels were notably increased in the D5 and D7 groups compared with those in D0, D1, and D3 treated groups (*p* < 0.05), while there were no significant differences among the D0, D1, D3 groups and between the D5, D7 groups (Table 7).

**Figure 9:**
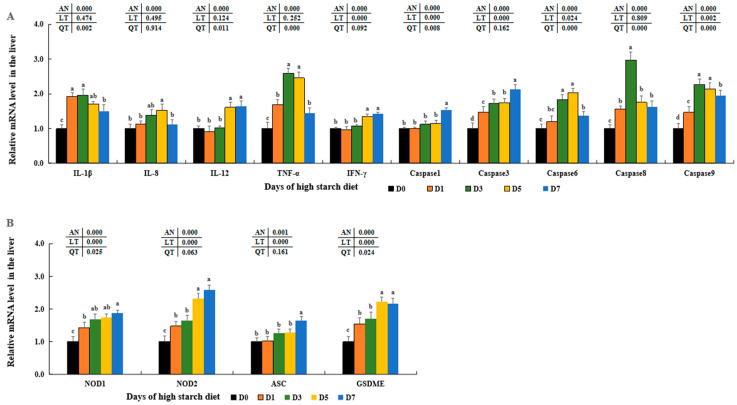
Effect of high-starch diet on related mRNA levels of hepatic inflammation in largemouth bass. (**A**) Inflammatory reaction, (**B**) The NLRP3 inflammasome activation. Data are reported as the mean ± SD of three replicates (*n* = 3). Bars with different superscripts (^a–d^) are significantly different (*p* < 0.05).

Expression levels of *IL-1β*, *IL-8*, and *TNF-α* were first increased and reached the maximum in the D3 and/or D5 groups compared to those in the D0 groups (*p* < 0.05) and then reduced in D7 groups. Although there were no differences in the D0, D1, and D3 groups (*p* >0.05), expression levels of *IL-12* and *IFN-γ* were notably increased in the D5 and D7 groups compared to those in the D0 groups (*p* < 0.05), with no significant differences observed among D5 and D7 (*p* > 0.05). In addition, transcription levels of apoptosis genes (*caspase 1* and *caspase 3*) were increased in the D5 and D7 groups, and both peaked in the D7 trial groups (*p* > 0.05). And the transcription levels of *caspase 6*, *caspase 8*, and *caspase 9* also first increased and reached peaks in the D5 and/or D3 groups compared to those in the D0 groups (*p* < 0.05) and then all decreased in D7 groups, with notable differences between D7 and D0 groups (*p* > 0.05) (Figure 9A). Moreover, the expression levels of NLRP3 inflammasome key molecules, including nucleotide-binding oligomerization domain-containing protein 1 (*NOD1*), *NOD2*, and gasdermin-E (*GSDME*), were notably elevated after consuming a high-starch diet, peaked in the fish liver of D7 or D5 groups compared to that of D0 groups (*p* < 0.05). Although there were no significant differences in the D0–D5 groups, apoptosis-associated speck-like protein-containing CARD (*ASC*) levels were notably elevated in the D7 groups (*p* < 0.05) (Figure 9B).

### 3.10. Fish Hepatic Histopathological Lesions

**Figure 10:**
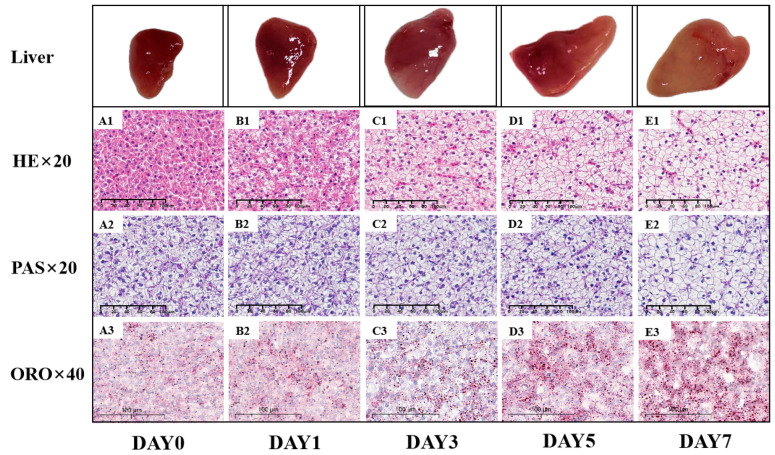
HE staining (magnification × 20), PAS staining (magnification × 20), and ORO staining (magnification × 40) of the liver sections of juvenile largemouth bass fed with high-starch diet containing D0 (**A1**–**A3**), D1 (**B1**–**B3**), D3 (**C1**–**C3**), D5 (**D1**–**D3**), and D7 (**E1**–**E3**).

The liver samples of juvenile largemouth bass in the high-starch group (B–E) and the control group (A) were observed by photographing, hematoxylin-eosin staining (HE), periodic acid-Schiff staining (PAS), and Oil Red O staining (ORO) (Figure 10). Within the D0 category (A1–A3), the livers of the largemouth bass displayed a reddish hue and no apparent abnormal characteristics, featuring well-formed hepatocytes with distinct nuclei. Additionally, the glycogen granules and lipid droplets in the liver cells of the largemouth bass were both smaller and of a lighter shade. Within the D1 groups (B1–B3), there were instances of pale livers displaying a dense nuclear phenotype and indistinct liver cords, often indicating the early stages of liver fibrosis in clinical settings. Additionally, there was an increase in glycogen granules and lipid droplets. In D3 (C1–C3), D5 (D1–D3), and D7 (E1–E3) groups, the white areas in the livers of largemouth bass increased and the shape became larger, and glycogen granules and lipid droplets were increased with culturing times with the high-starch diet.

## 4. Discussion

Previous studies have found dietary starch levels could modulate body indices (VSI, HSI, and ISI), which can also influence animal body compositions [41,42]. The current research found that high-starch diet (20%) led to a notable increase in VSI, HSI, and ISI in largemouth bass as the culturing days increased, similar with previous studies on giant gourami (*Osphronemus goramy*) [7], blunt snout bream (*Megalobrama amblycephala*) [43], and black carp (*Mylopharyngodon piceus*) [13]. Additionally, our findings indicated a significant rise in hepatic lipid levels over time due to high starch exposure, aligning with earlier studies on largemouth bass [44,45] and GIFT tilapia (*Oreochromis niloticus*) [5]. Higher dietary starch, when combined with these findings and our results, was found to induce hepatic lipid synthesis and accumulation in largemouth bass.

Hematological parameters have been important physiological assays for analyzing pathological variations in human and fish species [31,46,47]. Our results showed that high starch increased RBC counts and HGB levels, especially in the D1, D3 and D5 groups, which was in line with former results in Siberian sturgeon (*Acipenser baerii*) [48]. It is well-known that RBC and HGB undertake key roles in increasing oxygen-carrying, metabolic capacity and redox regulation in humans and animals [49,50,51]. Therefore, combining these findings and our results, it could be inferred that the largemouth bass needs more oxygen to carry out various physiological activities during the absorbing and metabolic processes of higher dietary starch. In addition, our results found high starch significantly increased counts of WBC, NEU, LYM, MON, EOS, and BAS with increasing culturing times, which agrees with previous results in *Labeo rohita* [52] and gilthead sea bream (*Sparus aurata*) [53]. It is well-known that adequate counts of WBC, NEU, LYM, MON, EOS, and BAS could improve immunity and enhance the defense effects when the animal is infected by some kinds of pathogens or fed with adequate nutrients [54]. However, excessive counts of WBC, NEU, MON, EOS, and BAS could induce inflammation and/or inflammatory responses in dairy goat [55], rat [56], and humans with type 2 diabetes mellitus [57]. The combination of these discoveries and our study outcomes indicates that increased consumption of dietary starch may lead to inflammation or inflammatory reactions in largemouth bass.

Serum biochemical indices are widely recognized as crucial indicators of health and metabolic status in both humans and animals. ALT and AST variations reflect hepatic metabolism and environmental abnormalities in animals [58]. Increased serum ALT and AST levels in the D3, D5, and D7 groups indicated that hepatic metabolism might be disturbed or damaged mediated by high dietary starch in largemouth bass [17]. Meanwhile, serum BUN variations could correlate with nitrogen utilization and reflect the homeostasis of exogenous amino acids [59]. So, higher BUN contents presented in D1–D7 groups indicated high dietary starch might impair exogenous nitrogen utilization in largemouth bass. Although ALP could act as an immune defense biomarker [60,61], excessive levels of ALP could couple degeneration, necrosis, and destruction of the liver due to cellular damage [62]. Moreover, higher levels of serum ALP are also correlated with the bile ducts obstructed in metabolic liver diseases [60]. Combined with higher serum ALP activities in fish fed high-starch diets in this study, it is suggested high dietary starch could cause cellular damage in largemouth bass. In addition to being an important immune index, serum ALB also plays a key role in the antioxidant function [63]. Considering the lower levels of serum ALB in D7 groups, it indicated that high dietary starch could impair immunity and increase the danger of oxidative stress in largemouth bass. Previous studies have found higher levels of serum TBA could be correlated with early-stage metabolic liver disease mediated by higher contents of cholesterol in animals [37,64]. Therefore, elevated levels of serum TBA suggest that increased dietary starch not only worsens liver damage but also triggers cholestatic disease in largemouth bass [65,66]. In typical physiological conditions, HDL-C moves total cholesterol from peripheral tissues to the liver for additional processing and bile acid production, whereas LDL-C transports hepatic cholesterol to other tissues [67]. Our findings showed contrasting trends in the HDL-C and LDL-C levels in D7 groups when compared with those in D0 groups (Table 4), which is consistent with previous findings in mice [68] and juvenile blunt snout bream [43]. Moreover, some researches have shown the level of TG and TC is positively proportional to the severity of fatty liver [67,69]. Combined with higher contents of serum TAG and TC, it is suggested that high dietary starch could not only prevent TC and TAG from being absorbed and utilized by the body, but also induce their accumulation in the body and then result in dyslipidemia and metabolic fatty liver [70].

Carnivorous fish are recognized as having weak capabilities for processing glucose and often present persistent hyperglycemia after feeding on a diet with high starch [2]. INS, ADPN, and GC play a crucial role as endocrine hormones in maintaining the glucose balance by managing fluctuations in serum glucose levels in both mammals and animals, as highlighted in studies by del sol Novoa et al. [71], Xu et al. [72], and Zhong et al. [17]. Elevated levels of serum glucose can trigger INS and ADPN release or decrease glucagon production to alleviate stress from hyperglycemia and maintain the glucose balance [73]. Our research discovered contrasting trends in the levels of serum INS, ADPN, and GC, despite a consistent increase in serum glucose levels in largemouth bass fed a high-starch diet. Likewise, earlier research has demonstrated that a 20% wheat starch diet led to a notable increase in blood glucose and INS levels in largemouth bass [6] and over a 4-week period in rainbow trout [74]. Conversely, it was observed that a high-starch diet resulted in significantly higher blood glucose levels after 3 h of feeding, but had no impact on INS levels in largemouth bass fed a diet with high starch (13.56%) [18]. As for these differences on the INS contents, it might be due to the different sample time in these studies. Furthermore, the levels of INS and ADPN were both decreased in the D7 groups, indicating that inadequate secretion of these hormones was the primary cause of glucose intolerance in carnivorous fish species [2]. In addition to reducing appetite signal, LEP also plays a crucial role in controlling fat or lipid reserves in both humans and animals [75,76,77]. Considering these findings and higher contents of serum LEP in this study, it indicated that higher starch could induce LEP secretion, which could then initiate the self-regulatory mechanism in response to higher serum glucose in largemouth bass [78]. Moreover, higher LEP levels were also presented in these obese individuals with a higher percentage of body fat, which was mainly mediated by typical LEP resistance in obesity [79]. The combination of these discoveries and our own results indicates that elevated starch levels may lead to LEP resistance, ultimately disrupting the regulation of lipid metabolism in largemouth bass, although further research about this resistance mechanism is needed to fully understand this.

As a major metabolic organ, the liver tissue plays important roles regulating glucose and lipid metabolic processes, include glycolysis, gluconeogenesis, glycogen synthesis, lipogenesis and lipolysis [10]. The important role of GLUT2 in regulating glucose transfer between the liver and serum through the PI3K/AKT signaling pathway has been widely recognized [80,81]. Our research found that elevated levels of dietary starch significantly increased GLUT2 levels at both transcription and protein levels, consistent with findings in rainbow trout (*Oncorhynchus mykiss*) [82], blunt snout bream [83], and Gibel carp (*Carassius gibelio*) [84]. This indicates that high starch intake may enhance the transport of plasma glucose into hepatic cells by stimulating GLUT2 in largemouth bass. Numerous past research has indicated that elevated levels of starch can trigger glycolysis pathways through the INS signal pathway in mice [85], blunt snout bream [86], common carp (*Cyprinus carpio*) [87], and largemouth bass [45]. Levels of GCK, PFK, PK, *ISRa*, *ISRS2b*, *PI3Kc*, and *AKT1* were significantly increased in the D5–D7 groups that were fed the high-starch diet, as well as with higher PA contents in our findings. Our results, along with these discoveries, suggest that elevated levels of starch may enhance glucose metabolism by stimulating glycolysis through the activation of the typical PI3K/AKT signaling pathway in largemouth bass. Additionally, gluconeogenesis was also stimulated with elevated levels of FBP and G6Pase in the D5–D7 groups that were given high-starch diet, which is similar with former results in largemouth bass fed 13.56% dietary starch [18], suggesting there was a competition between dietary glucose and endogenous productive glucose. Based on these findings, it was suggested that high levels of starch could lead to glucose metabolic disorders and hinder glucose utilization in largemouth bass.

Furthermore, the hepatic production of glycogen in animals may be controlled by the PI3K/AKT signaling pathway, which acts on its downstream proteins and enzymes (*GYG2*, GCS, GBE, and GDE) as indicated by Yang et al. [37] and Zhang et al. [88]. Typically, increased levels of *GYG2*, GCS, and GBE may play important functions in enhancing glycogen production, whereas elevated GDE levels could facilitate the breakdown of hepatic glycogen [2]. Past research has shown that the amounts of glycogen in the liver increased as the levels of starch in the diet rose for red spotted grouper (*Epinephelus akaara*) [89] and blunt snout bream [90]. Furthermore, by utilizing [U-14C] glucose as a marker, it showed a significant rise in glycogen synthesis from glucose in largemouth bass when the dietary starch level was raised from 5% to 15% [91]. Combined with these findings, it indicated that high starch may induce hepatic glycogen synthesis in largemouth bass through the PI3K/AKT signal transduction pathway, as evidenced by the variations of these key molecules, LAG contents, and PAS-stained histological sections in our results. Nevertheless, levels of hepatic LA and LDH activities in serum and liver showed significant increases in the D1–D7 groups, mirroring findings in goats [92] and cattle [93] that were given a high-starch diet. Past research has shown that increased LA accumulation can lead to risks such as lactic acidosis, nonalcoholic fatty liver disease, and tumors in humans and animals [94,95,96,97]. Based on these discoveries and our findings, it appears that excessive LA levels could lead to lactic acidosis and metabolic disorders in largemouth bass fed a diet high in starch.

Typically, Ac-CoA is converted by ACC1 into Mal-CoA, which is then transformed by FAS into fatty acids (FAs) within the liver cells [18,98]. The citrate-pyruvate pathway, facilitated by ME, and the pentose-citrate pathway, facilitated by *G6PD*, supply the necessary energy for this process [37]. Consistent with prior findings in largemouth bass that were given a high-starch diet [18], we also observed elevated levels of ACC, FAS, Mal-CoA, *SCD*, *ME1*, *G6PD*, and *PGD* in fish fed a high-starch diet. This suggests that high starch intake can stimulate fatty acid production by up-regulating the expression of these genes associated with lipid synthesis in the liver of largemouth bass. Moreover, *FATP1* can facilitate the transfer of FA to the endoplasmic reticulum within liver cells for the production of LTAG, as demonstrated by Ipsen et al. [99] and López [100]. And *GK*, *GPAT3*, *GPAT4*, and *DGAT2* are the rate-limiting enzymes during LTAG synthesizing processes [101]. Then, these synthesized LTAG could bind apolipoprotein B100 (*APOB100*) and enter the bloodstream by free diffusion [102]. Meanwhile, *Plin2*, as a key lipid droplet coated protein, is tightly related with lipid droplet biogenesis and storage in tissues [103]. Elevated levels of *Plin2* are often associated with various metabolic disorders, including insulin resistance and type 2 diabetes in human and animals [103,104]. The study found that *FABP10*, *GPAT3*, *GPAT4*, *DGAT2*, *APOB100*, and *Plin2* levels were notably higher in groups consuming high-starch diets, consistent with earlier findings in largemouth bass [18] and gilthead sea bream [105]. Previous research has shown that the AKT/SREBP1c signaling pathway can stimulate LTG synthesis in both animals and fish, as documented by Wang et al. [106] and Ferre et al. [107]. In addition to increased levels of these important molecules, this study also showed that high starch in largemouth bass can activate the AKT/SREBP1c signal pathway, leading to enhanced FA and LTAG synthesis, as well as lipid droplet formation, as demonstrated by LTAG levels and histological sections stained with HE and ORO in this study.

*SREBP2*, a crucial transcription factor, has the ability to regulate the metabolism and balance of cholesterol in the liver by influencing the transcription of genes that are regulated by sterols, such as *AACS, HMGCS*, *HMGCRa*, and *CYP51* [18,19]. *AACS* has the ability to use ketone bodies for the creation of cholesterol and fatty acids, converting acetoacetate into its CoA ester to generate acetoacetyl-CoA in the cytosol [108]. This process is facilitated by *HMGCS*, which transforms acetoacetyl-CoA into 3-hydroxy-3-methylglutaryl-CoA (HMG-CoA) [109,110]. Subsequently, *HMGCRa* is able to facilitate the transformation of HMG-CoA into mevalonic acid in order to generate cholesterol [111]. *CYP51* plays an essential role in the sterols’ production and is a significant focus of cholesterol-lowering medications according to recent studies [112,113,114]. Our results showed that a diet with high-starch significantly increased the levels of *SREBP2* and its target genes related to cholesterol synthesis (*AACS*, *HMGCS*, *HMGCRa*, and *CYP51*) in groups D1, D3, and D5. This effect was consistent with findings in largemouth bass [18] and male C57BL/6J mice [115], suggesting that high starch intake may stimulate cholesterol production through the *SREBP2* signaling pathway in the hepatic cells of largemouth bass. The expression of these genes was significantly reduced in the D7 groups compared to that in the D3 groups, which might be due to the negative feedback regulatory mechanism mediated by higher contents of TC in the liver of largemouth bass [19,20], although further research is needed to fully understand this regulatory process. In addition, *SOAT2* plays an important function in lipid droplet biogenesis and storage by converting cholesterol to cholesteryl ester in cells [104,116]. Prior research has shown that an increase in lipid droplets can be triggered by elevated glucose levels in normal colon cells and colorectal cancer stem cells [117], as well as in mice with type 2 diabetes [118]. Based on these results, it is proposed that increased levels of *SOAT2* may be responsible for the accumulation of lipid droplets in largemouth bass fed a high-starch diet. Meanwhile, *ABCG5* and *ABCG8* act as key proteins transporting endogenous cholesterol out of the liver into other tissues, while *NPC1L1* plays key roles in up-taking intestine cholesterol into hepatic cells [119,120]. Combined with these findings and lower levels of *ABCG5*, *ABCG8*, and *NPC1L1* in this study, it indicated these three key reduced transporters might mediate the hepatic cholesterol decomposition in largemouth bass [116].

In general, bile acid synthesis is mainly regulated by two key rate-limiting enzymes (*CYP7A1* and *CYP27A1*) mediating the oxidation of cholesterol in hepatocytes [120,121]. Higher *CYP7A1* and *CYP27A1* levels in D1–D3 groups indicated high starch could induce bile acid synthesis via the oxidation of cholesterol in hepatocytes of largemouth bass. The reduced levels of *CYP7A1* and *CYP27A1* in D5–D7 groups might be caused by the negative feedback regulatory mechanism mediated by higher TBA contents in hepatocytes [121,122]. Furthermore, liver bile acids may be moved into the bile canaliculus using the bile salt transporter known as the canalicular bile salt export pump (*BSEP*) [123,124]. Although there were no marked differences among D0–D5 groups, lower *BSEP* levels in D7 groups indicated high starch might block the transportation of hepatic bile acids into bile canaliculus and then result in possible cholestasis [120,121]. Two important transporting polypeptides, *NTCP* and *OATP1*, facilitate the absorption of bile acids from the portal vein into hepatocytes [125]. Higher *NTCP* and *OATP1* levels in D1–D5 groups indicated that bile acids could be taken up into hepatocytes induced by high starch, which might further aggravate cholestasis in largemouth bass. In humans and rodents, studies have found higher levels of hepatic *OST-α* could help bile acid excretion into the circulating system [122,126]. Combined with higher *OST-α* mRNA levels and serum TBA contents, it indicated that high starch could drive bile acid excretion into the circulating system via a heightening expression of hepatic *OST-α* in largemouth bass, although this driving mechanism needed to be studied in fish species [127].

It is well known that cellular ROS is mainly generated by NADPH oxidases (*NOX*) and the respiratory electron transport chain (ETC) of mitochondria during glucose metabolic processes in animals [28,128,129]. Our study revealed that the expression of certain genes, such as *G6PD*, *NOX1*, NADPH oxidase activator 1 (*NOXA1*), and cytochrome c oxidase 4 (*COX4*), along with the levels of H_2_O_2_ significantly increased with high-starch diets. This is consistent with findings in humans with diabetes [23,25,130], suggesting that high starch intake may enhance oxidative phosphorylation, leading to elevated ROS levels in largemouth bass liver. In general, SOD and CAT in antioxidant systems could catalyze ROS and reduce oxidative stress in animals [5,21]. Meanwhile, increased GST and MDA amounts can symbolize physiological biomarkers to oxidative or inflammatory stress in cultured animals [1,3]. Past research has shown that the antioxidant abilities were diminished by elevated starch levels in black carp [131], black sea bream (*Acanthopagrus schlegelii*) [12], GIFT tilapia [5], and largemouth bass [132]. Similarly, our results also found a notable reduction in the amounts of SOD, CAT and T-AOC along with a significant increase in the amounts of GST, MDA, and H_2_O_2_ in the D5 and D7 groups that were given a high-starch diet. Moreover, a specialized thioredoxin system within mitochondria, consisting of *Trx2*, *TrxR2*, and *Prx3*, has been identified as crucial for protecting against oxidative stress induced by H_2_O_2_ through disulfide reductase functions [133,134]. Our results show that a high-starch diet could inhibit the genetic expressions of *Trx2*, *TrxR2*, and *Prx3* in the liver in largemouth bass. Considering these findings and our data, it indicated that high starch could aggravate ROS damage and disturb mitochondrial redox homeostasis by decreasing antioxidant capabilities in largemouth bass [128].

**Figure 11:**
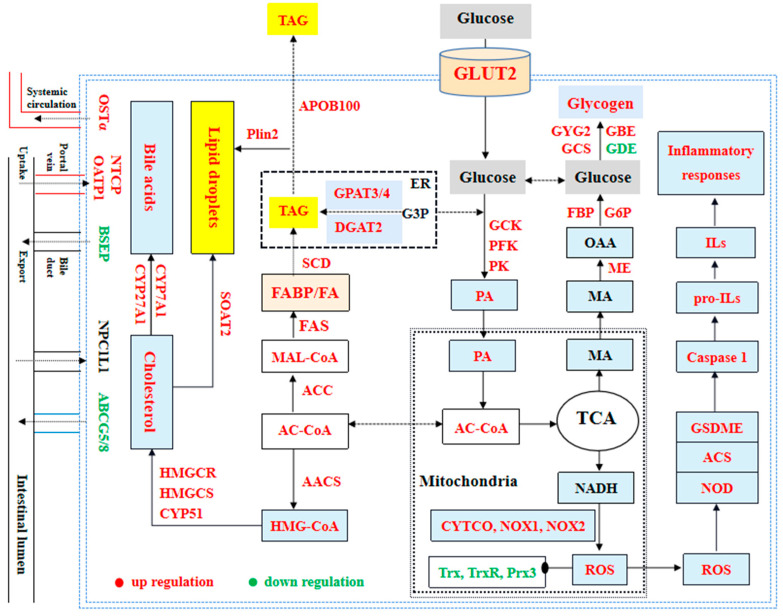
The summary of glucose and lipid metabolism, antioxidant and oxidative responses, and inflammatory responses in the hepatic cells of largemouth bass.

Numerous research studies have shown that an overabundance of *NOX*-derived ROS can lead to inflammation by boosting the generation of pro-inflammatory cytokines in the liver, such as IL-1β, TNF-α, IFN-γ, IL-6, IL-8, IL-12, IL-17, and others [24,29]. Prior studies have discovered elevated amounts of inflammatory markers such as IL-1β, TNF-α, and IFN-γ, which were presented in human with diabetes [135,136,137], pig with malnutrition [138], and mice fed high-carbohydrate diets [139]. Likewise, our results showed elevated concentrations of IL-1β, IL-6, IL-8, IL-12, IL-17, TNF-α, and IFN-γ in the hepatic cells of largemouth bass with increasing time of feeding high-starch diet in this study. Combined with previous results and our findings, it further proved that inflammatory responses could be caused by ROS excessively generated during the metabolism of higher dietary starch in largemouth bass. In addition, the secretion and maturation of these pro-inflammatory cytokines were mediated by the typical NLRP3 inflammasome in human and animal cells [24,33]. The NLRP3 inflammasome consists of the NLRP3 sensor molecule, the *ASC* adaptor protein, and pro-caspase-1 [35]. Various molecular and cellular events, including ROS overload, can lead to the stimulation of the NLRP3 inflammasome [27,140]. In the formation of NLRP3 inflammasomes, pro-caspase-1 can transform into cleaved-caspase-1, triggering the processing of pro-IL-1β precursors into their active states [135,141]. Recent research has shown that elevated glucose levels can stimulate the generation of ROS, boost the triggering of the NLRP3 inflammasome, and increase IL-1β release in macrophages [34,142] as well as in RSC96 (rat Schwann cell line) cells [27]. Moreover, additional research has shown that elevated glucose levels can lead to the depletion of retinal pericytes through a process of inflammatory cell death involving NLRP3 and caspase-1 [141]. The research also revealed increased *ASC*, *NOD1*, *NOD2*, *GSDME*, and caspase-1 expression in fish groups exposed to high starch, mirroring findings in human and rat cells [27,34]. By combining these discoveries with our own results, we can conclude that inflammatory reactions may be initiated by the activation of the NLRP3 inflammasome induced by ROS overload in largemouth bass fed with high starch (Figure 11).

## 5. Conclusions

In summary, our results found high starch could increase body physical indicators (HSI, VSI, and ISI) and counts of leukocytes (WBC, NEU, MON, EOS, and BAS). And high starch could cause insufficient secretion of INS and ADPN and then induce glucose metabolic disorders and abnormal glycogen accumulation. Meanwhile, high starch could cause abnormal TAG and cholesterol accumulation via *SREBP1/2* signal pathways. In addition, high starch could induce oxidative stress by heightening ROS contents and reducing antioxidant capabilities. Oxidative stress or ROS overload induced by high starch in largemouth bass can activate the NLRP3 inflammasome, leading to inflammatory responses (Figure 11).

## Figures and Tables

**Table 1:** Composition and nutrient levels of experimental diets (air-dry basis).

Ingredient	Composition of Diets (%)	
	Diet 1	Diet 2
Fish meal ^a^	20.00	20.00
Soybean meal ^b^	10.00	10.00
Casein ^c^	35.00	35.00
Pregelatinized tapioca starch ^d^	0.00	20.00
Rapeseed oil ^e^	6.50	6.50
Lecithin ^f^	2.00	2.00
Mineral premix ^g^	2.00	2.00
Vitamin premix ^h^	1.00	1.00
Choline chloride ^i^	0.40	0.40
Microcrystalline cellulose ^j^	23.10	3.10
Nutrient levels (%)		
Moisture	7.31	7.18
Crude protein	46.63	46.74
Crude lipid	10.85	10.55
Ash	6.33	6.43

^a^ Supplied by Zhejiang Dongyu Biotechnology Co., Ltd. (Huzhou, China). ^b^ Supplied by Ningbo Food Co., Ltd. (Ningbo, China). ^c^ Obtained from Gansu Hualing Dairy Co., Ltd. (Lanzhou, China). ^d^ Supplied by Xinxin biochemical technology Co., Ltd. (Hangzhou, China). ^e^ Rapeseed oil was produced using oil press. ^f^ Supplied by Jiangsu Yuanshengyuan Biological Engineering Co., Ltd. (Nanjing, China). ^g^ Mineral premix (mg kg^−1^ diet): Ca(H_2_PO_4_)_2_, 12,000 mg; KI, 0.4 mg; CoCl_2_·6H_2_O, 52 mg; CuSO_4_·5H_2_O, 16 mg; FeSO_4_·7H_2_O, 200 mg; ZnSO_4_·7H_2_O, 280 mg; MnSO_4_·H_2_O, 45 mg; MgSO_4_·7H_2_O, 1200 mg; NaCl, 60 mg. ^h^ Vitamin premix (mg kg^−1^ diet): vitamin A, 35 mg; vitamin D, 6 mg; vitamin C, 1000 mg; vitamin E, 300 mg; thiamine, 30 mg; riboflavin, 50 mg; pyridoxine hydrochloride, 20 mg; vitamin B_12_, 0.1 mg; vitamin K_3_, 10 mg; inositol, 800 mg; pantothenic acid, 60 mg; folic acid, 20 mg; niacin, 200 mg; biotin, 60 mg. ^i^ Supplied by Zhejiang Yixing Feed Group Co., Ltd. (Jiaxing, China). ^j^ Supplied by Sinopharm Chemical Reagent Co., Ltd. (Shanghai, China).

**Table 2:** The list of PCR primers used in this study.

Gene	Forward (5′-3′)	Reverse (5′-3′)	Reference
*GLUT2*	TCACCGTGTTTATTTATCTTCG	AGCTCCGTATCGTCTTTGG	XM_038728860
*GCK*	AAGGGAACAATGTTGTGGG	AGCTGCGGTCCTCGTAAT	XM_038703172
*PFK*	CTGTATAATCCCTGCCACCAT	TCTCCACCACAAACACTCG	XM_038720351
*PK*	CACCAACCCATTCATTTGC	GTGTCATCACCTCAGAGTAGCG	XM_038711316
*LDH*	GCAGGAGGGTGAAAGCC	GGTTGGAGACCACGATGAGT	XM_038726015
*FBP*	CTGCGGCTGCTGTATGAA	CCCGCTGATGGATGCTCT	XM_038704229
*G6Pase*	GTCAACAAGCAACCCAACG	AGTGAGGACACCACGACCC	XM_038734911
*ISR*	CCCGAGACATCTATGAGACCG	CAAAGCACGACGCCAAAT	XM_038715865
*ISRS2b*	TCCCCGTTCACACTCCTCT	CATTTTTGTTCCGCACCAC	XM_038720730
*PI3Kc*	GCCAGTAGTGAGCAGTGAAAGC	TCCATGACGGCATAGATAGCA	XM_038693595
*AKT1*	AGCGGGCTCGGTTCTACGGT	ATCTTTGTCCAGCATGAGGTTT	MG993041
*SREBP1c*	GAGGACACCAAGCCGAATG	TGCCAGAGGGTTGAGGGA	XM_038699585
*G6PD*	TGGTGCTGGGTCAGTATGT	GCGCAGGTAAAGGTGGC	XM_038722146
*PGD*	TTTGTGGTCTGCGCTTACA	GATCCTCCTGGGCTTCTTC	XM_038739743
*GYG2*	GATAGCCAAGGAACAGCC	TGAAGCATCTTAGAACGGAGT	XM_038722829
*GCS*	CATCCAACGGACACGAAC	CATCTTGAACTTGTCAGGGA	XM_038695255
*GBE*	TTGACGGCTTCCGATTT	ATTGGCAAGCATCAGGTA	XM_038694127
*PP1*	GGCTGTTTGAGTATGGTGGC	TCTGGGTATTTGATCTTGTAGGC	XM_038728851
*GDE*	TCCTTGATGTAACCCACGAC	CAGTAGCACAGCAAGCCATA	XM_038718921
*ACC*	GCCAGTCTCCCAACTCCTA	ATGCGATACCTGTCCACCT	XM_038709728
*FAS*	CATTCCGTAGTAGGATAAGTCAACA	CATAGTCATAACCACGCAGTCG	XM_038735140
*SCD*	CCCTTCAGCATCTCCTTT	GTGGTAATGTGGCCTTGTA	XM_038735580
*ME1*	TCGCTAAGGAGGAGTGTTTG	GTTTCTTGATCTGTGGGTGC	XM_038712129
*FABP-L*	GGTCAAGTCGGTGGTTCA	ATGCGTTTGCTTGTCCTC	XM_038704629
*FATP4*	GATTCTACCGTTCATCTACCC	GAATGATCCTGCCGACTA	XM_038733650
*GK*	TGCGGACTCAATCAACG	TCCATCAGCCAGCGTAG	XM_038720816
*GPAT3*	AGAGGGCGATAGTGAGGG	CAGGATGGGAAGTTTGGTC	XM_038733141
*GPAT4*	CTGTTGTTGGGCTGTTGC	TCCATTCTTGGGTTTATTCTC	XM_038704699
*DGAT2*	CTTCCGCTTGCCCGTCCTT	GCATTTCCTGTCCCGTTAT	XM_038708163
*Plin2*	GAGTGGACACGGCCCTAA	GAACCCAGGCGGACATAG	XM_038730762
*APOB100*	GATTGTAAAGTTTGAGGCTGAC	TGCATGAATTTCGTAGGG	XM_038727548
*SREBP2*	TGTTGCCGTGGGTGATG	TTGCGATGCCTCCAGAAT	XM_038694760
*AACS*	GCTCGGCTCAACTACGCT	CGAACAACGCCACATCTT	XM_038703519
*HMGCRa*	GGTGGAGTGCTTAGTAATCGG	CACGCAGGGAAGAAAGTCA	XM_038702603
*HMGCS*	CCTGGACGACTTTGGCTAT	CCTGTGAAGGGTCCTGTCT	XM_038733206
*CYP51*	TCGCCTCAGACTGTAGCAG	GAGGTAGCGGTCAGGGTT	XM_038725400
*SOAT2*	CTTGGCAATGGGCTTCGG	GCTCTGTCGCTTGTCGTTC	XM_038695616
*ABCG5*	ACGGATGTAGGGAGGGAC	CGCTTCTTGTAGGAGGGTA	XM_038737039
*ABCG8*	GCTTTCTCATGCCTCCTTTA	GTTCATCGCCTCCACCAC	XM_038737035
*NPC1L1*	CCTTCCCTCCTCGCTGAT	CCAAGAACACGCCCAATC	XM_038702470
*CYP7A1*	CGGCGGTTGCGTTACTT	GATAGCAGGGTCCAATAGTTC	XM_038717160
*CYP27A1*	TTGCCTCTATGCCATCAGTC	TCACTCCGAAGCCAAACG	XM_038720051
*BSEP*	AGATGCTCCGTACCAAGCG	CACCAAGTAACCTCCAAACCTAT	XM_038716782
*NTCP*	CAAGGCTGTCGGAGGCAACG	ATGGAGGAGAAGGGAACG	XM_038732638
*OATP1*	GCAGTGGCAGTTGGGATC	GCAGCAGCAGAAGGAGGTAT	XM_038738452
*OST-* *α*	CCAAGAAGACCACCATCA	TACACCACGACTGCAAAA	XM_038708198
*MRP3*	ACGTGGAGTTCCGCGACTA	GTGCGACCAACGATACCAA	XM_038719452
*Cu/Zn-SOD*	TGAGCAGGAGGGCGATTC	GCACTGATGCACCCATTTGTA	XM_038708943
*Mn-SOD*	CAGGGATCTACAGGTCTCATT	ACGCTCGCTCACATTCTC	XM_038727054
*CAT*	ACCTATTGCTGTCCGCTTCTC	TCCCAGTTGCCCTCCTCA	XM_038704976
*GST1*	GAGCCCATCAGAACACCC	ACCCAAATAGCACCCAAC	XM_038711179
*Trx2*	TTCAGGACCACGATGACTTCAC	TGCAACAGCCTTCTCCAACC	XM_038693856
*TrxR2*	GCCTTCTGTAGAGGGAGACA	CTGAACCACCACCAATGAC	XM_038703974
*Prx3*	ACAAAGCCAATGAGTTCCA	TTGCCTAAGCCTCCAGTC	XM_038737170
*COX4*	GCCTGACAAACGCTACAAAG	GGTACAAGGCAATCTTCTCCTC	XM_038723148
*NOX1*	GTGTCCCATCCCTCAGTT	GCCGCATCACAATCTTC	XM_038698886
*NOXA1*	TCCTACACTGCCACCTATGC	TGGACCTTCACCACCACAG	XM_038704394
*HO1*	TCTTTGGCGTAAACTGGAGG	TAGCGAGTGTAGGCGTGGG	XM_038694281
*TNF-α*	CAACGGCAAGTGTCAAACCC	TCTTGTCCTGAGCCCTTGGTAT	XM_038723994
*IL-1β*	GCGACCGCAGTAAGAAAG	CAGACGGGATAGTCGATGTA	XM_038733429
*IL-8*	TTCTCCTGGCTGCTTTGG	TGGATGGCCCTCCTGTTA	XM_038704093
*IL-12*	CCGCTGTTATTCAGTCTTACC	GCATCAGGGAGCAGTTCA	XM_038693841
*IFN-* *γ*	GAGTTGCTTTGGCGTTTG	TGTTGATGCTCCTGGTGA	XM_038709291
*Caspase1*	ATGAAGCCAGCAGGAAGC	TTGGGATGAGTGCGTTTG	XM_038694367
*Caspase3*	CGTGGTACAGACCTGGATG	GCCTGGAGCAGTGGAATA	XM_038699323
*Caspase6*	ATGCCGTGGACAGTGAGTT	CATACCAGGACCCGTTGAT	XM_038710174
*Caspase8*	CAGGCTCCATCTACATCC	TCCCTTGCTGACCTCC	XM_038718636
*Caspase9*	TCCCAGTTCAGCACATCA	GGACCTCATTAGGCGACAC	XM_038734900
*NOD1*	TGTTGGTGGGAGGTATTTG	TGGTAAGACGTGGGTGGT	XM_038712291
*NOD2*	GGGCAATAAGATAGGTGATG	TGATAATGTTGGCGAGGG	XM_038701062
*ASC*	AAATAAGGTGGAGGGTAA	AGTCTGCTTCACAGTGGC	XM_038710753
*GSDME*	ACATGACGGACGCTACGA	GCTGAAAGGTGCTGGAAA	XM_038711654
*β* *-actin*	TTCACCACCACAGCCGAAAG	TCTGGGCAACGGAACCTCT	XM_038695351

*GLUT2*, glucose transporter 2; *GCK*, glucokinase; *PFK*, phosphofructokinase; *PK*, pyruvate kinase; *LDH*, lactate dehydrogenase; *FBP*, fructose-1,6-bisphosphatase; *G6Pase*, glucose-6-phosphatase; *ISR*, insulin receptor; *IRS2b*, insulin receptor substrate 2b; *PI3Kc*, phosphatidylinositol 4,5-bisphosphate 3-kinase catalytic; *AKT1*, AKT serine/threonine kinase 1; *SREBP1c*, sterol regulatory element-binding protein 1c; *G6PD*, glucose-6-phosphate 1-dehydrogenase; *PGD*, 6-phosphogluconate dehydrogenase; *GYG2*, glycogenin 2; *GCS*, glycogen synthase; *GBE*, 1,4-alpha-glucan-branching enzyme; *PP1*, serine/threonine-protein phosphatase PP1; *GDE*, glycogen debranching enzyme; *ACC*, acetyl-CoA carboxylase; *FAS*, fatty acid synthase; *SCD*, acyl-CoA desaturase; *ME1*, NADP-dependent malic enzyme 1; *FABP-L*, fatty acid-binding protein liver type; *FATP4*, long-chain fatty acid transport protein 4; *GK*, glycerol kinase; *GPAT3*, glycerol-phosphate acyltransferase 3; *GPAT4*, glycerol-phosphate acyltransferase 4; *DGAT2*, diacylglycerol O-acyltransferase 2; *Plin2*, perilipin 2; *APOB100*, apolipoprotein B 100; *SREBP2*, sterol regulatory element-binding protein 2; *AACS*, acetoacetyl-CoA synthetase; *HMGCRa*, 3-hydroxy-3-methylglutaryl-Coenzyme A reductase; *HMGCS*, hydroxymethylglutaryl-CoA synthase; *CYP51*, lanosterol 14-alpha demethylase; *SOAT2*, sterol O-acyltransferase 2; *ABCG5*, ATP-binding cassette sub-family G member 5; *ABCG8*, ATP-binding cassette sub-family G member 8; *NPC1L1*, Niemann-Pick C1-like protein 1; *CYP7A1*, cholesterol 7-alpha-monooxygenase; *CYP27A1*, sterol 26-hydroxylase; *BSEP*, bile salt export pump; *NTCP*, sodium/taurocholate cotransporting polypeptide; *OATP1*, solute carrier organic anion transporter family member 1; *OST-α*, organic solute transporter subunit alpha; *MRP3*, canalicular multispecific organic anion transporter 2; *Cu/Zn-SOD*, Cu/Zn-superoxide dismutase; *Mn-SOD*, Mn-superoxide dismutase; *CAT*, catalase; *GST1*, glutathione S-transferase 1; *Trx2*, thioredoxin 2; *TrxR2*, thioredoxin reductase 2; *Prx3*, peroxiredoxin 3; *COX4*, cytochrome c oxidase; *NOX1*, NADPH oxidase 1; *NOXA1*, NADPH oxidase activator 1; *HO1*, heme oxygenase-1; *TNF-α*, tumor necrosis factor alpha; *IL-1β*, interleukin-1 beta; *IL-8*, interleukin 8; *IL-12*, interleukin 12; *IFN-γ*, interferon-gamma; *Caspase1*; *Caspase3*; *Caspase6*; *Caspase8*; *Caspase9*; *NOD1*, nucleotide-binding oligomerization domain-containing protein 1; *NOD2*, nucleotide-binding oligomerization domain-containing protein 2; *ASC*, apoptosis-associated speck-like protein containing a CARD; *GSDME*, gasdermin-E.

**Table 3:** Effects of dietary days on hematological parameters in largemouth bass fed high starch (mean ± SD, *n* = 15).

Items	Days of High Starch Diet							
	D0	D1	D3	D5	D7	AN	LT	QT
RBC ^A^	3.34 ± 0.10 ^b^	3.61 ± 0.19 ^ab^	3.61 ± 0.25 ^a^	3.60 ± 0.18 ^ab^	3.52 ± 0.17 ^ab^	0.028	0.461	0.061
HGB ^B^	117.13 ± 4.32 ^c^	134.88 ± 5.08 ^a^	133.00 ± 5.88 ^a^	136.63 ± 3.93 ^a^	126.00 ± 5.24 ^b^	0.000	0.031	0.154
WBC ^C^	129.75 ± 6.27 ^b^	160.97 ± 13.91 ^a^	160.22 ± 22.52 ^a^	163.54 ± 7.90 ^a^	175.13 ± 11.92 ^a^	0.000	0.000	0.001
NEU ^C^	2.71 ± 0.30 ^e^	5.20 ± 0.53 ^d^	6.90 ± 0.42 ^c^	7.68 ± 0.48 ^b^	8.67 ± 0.27 ^a^	0.000	0.000	0.000
LYM ^C^	129.55 ± 5.67 ^c^	135.88 ± 13.28 ^bc^	145.88 ± 16.04 ^ab^	149.59 ± 7.37 ^a^	147.65 ± 5.05 ^a^	0.002	0.000	0.000
MON ^C^	4.79 ± 0.19 ^d^	10.44 ± 0.21 ^c^	11.56 ± 0.25 ^b^	12.03 ± 0.49 ^b^	13.24 ± 0.55 ^a^	0.000	0.000	0.000
EOS ^C^	0.14 ± 0.02 ^c^	0.18 ± 0.01 ^c^	1.27 ± 0.13 ^b^	1.59 ± 0.04 ^b^	2.12 ± 0.40 ^a^	0.000	0.000	0.000
BAS ^C^	0.14 ± 0.01 ^d^	0.31 ± 0.07 ^d^	3.08 ± 0.01 ^c^	4.15 ± 0.09 ^b^	6.53 ± 0.39 ^a^	0.000	0.000	0.000
PLT ^C^	825.80 ± 47.56 ^a^	448.75 ± 49.66 ^b^	309.88 ± 57.81 ^c^	258.38 ± 50.96 ^cd^	223.63 ± 34.89 ^d^	0.000	0.000	0.000

Note: ^a–e^ Values in the same row with different letters indicate significant differences (*p* < 0.05). AN: ANOVA, LT: linear trend, QT: quadratic trend. A = 10^12^/L, B = g/L, C = 10^9^/L.

**Table 4:** Effects of dietary days on biochemical and hormone indicators of serum in largemouth bass fed high starch (mean ± SD, *n* = 9).

Items	Days of High Starch Diet							
	D0	D1	D3	D5	D7	AN	LT	QT
GLU ^A^	4.39 ± 0.06 ^d^	5.59 ± 0.06 ^c^	5.73 ± 0.32 ^c^	6.71 ± 0.14 ^b^	8.76 ± 0.39 ^a^	0.000	0.022	0.000
HDL-C ^A^	2.07 ± 0.12 ^b^	2.60 ± 0.06 ^a^	2.78 ± 0.10 ^a^	2.51 ± 0.15 ^a^	2.79 ± 0.13 ^a^	0.000	0.012	0.098
LDL-C ^A^	1.42 ± 0.05 ^b^	1.85 ± 0.08 ^a^	1.94 ± 0.05 ^a^	1.90 ± 0.10 ^a^	2.03 ± 0.09 ^a^	0.000	0.001	0.038
TG ^A^	5.18 ± 0.11 ^d^	6.44 ± 0.30 ^c^	7.32 ± 0.17 ^b^	9.17 ± 0.42 ^a^	9.00 ± 0.27 ^a^	0.000	0.000	0.506
TC ^A^	5.99 ± 0.42 ^c^	7.34 ± 0.20 ^d^	7.91 ± 0.08 ^a^	8.07 ± 0.07 ^a^	8.12 ± 0.25 ^a^	0.000	0.043	0.166
AST ^B^	56.10 ± 6.50 ^b^	84.77 ± 2.47 ^a^	90.00 ± 3.37 ^a^	85.33 ± 6.61 ^a^	97.70 ± 6.87 ^a^	0.000	0.002	0.096
ALT ^B^	3.73 ± 0.15 ^c^	6.90 ± 0.26 ^b^	8.17 ± 1.10 ^ab^	9.37 ± 2.15 ^a^	9.05 ± 0.35 ^a^	0.001	0.000	0.008
TBA ^C^	8.10 ± 0.46 ^d^	9.10 ± 0.10 ^c^	11.20 ± 0.44 ^a^	10.57 ± 0.25 ^ab^	10.00 ± 0.20 ^b^	0.000	0.015	0.000
ALP ^B^	104.77 ± 5.08 ^d^	138.50 ± 2.88 ^b^	156.47 ± 3.58 ^a^	143.57 ± 5.14 ^b^	114.80 ± 4.93 ^c^	0.000	0.715	0.000
ALB ^D^	8.27 ± 0.12 ^c^	10.50 ± 0.20 ^a^	10.10 ± 0.10 ^b^	9.80 ± 0.10 ^b^	7.20 ± 0.26 ^d^	0.000	0.026	0.222
LDH ^B^	751.93 ± 23.11 ^d^	1037.03 ± 28.85 ^b^	1398.83 ± 20.26 ^a^	941.77 ± 24.41 ^c^	925.3 ± 27.77 ^c^	0.000	0.754	0.001
BUN ^A^	0.99 ± 0.03 ^c^	1.66 ± 0.06 ^a^	1.55 ± 0.04 ^a^	1.21 ± 0.08 ^b^	1.11 ± 0.08 ^b^	0.000	0.272	0.501
INS ^E^	20.18 ± 0.88 ^c^	22.09 ± 0.32 ^b^	23.76 ± 0.49 ^a^	19.13 ± 0.19 ^d^	18.51 ± 0.47 ^d^	0.000	0.048	0.003
GC ^F^	9.17±0.06 ^c^	8.71±0.06 ^d^	7.70±0.08 ^e^	9.42±0.08 ^b^	10.05±0.03 ^a^	0.000	0.054	0.000
ADPN ^G^	57.18 ± 1.29 ^c^	63.85 ± 1.60 ^b^	84.15 ± 2.29 ^a^	66.42 ± 2.74 ^b^	66.27 ± 2.89 ^b^	0.000	0.725	0.167
LEP ^G^	0.12 ± 0.01 ^e^	0.24 ± 0.01 ^d^	0.31 ± 0.01 ^c^	0.49 ± 0.01 ^a^	0.37 ± 0.03 ^b^	0.000	0.000	0.000

Note: ^a–e^ Values in the same row with different letters indicate significant differences (*p* < 0.05). A = mmol/L, B = U/L, C = umol/L, D = g/L, E = mU/L, F = pg/ml, G = µg/L.

**Table 5:** Effects of dietary days on glucose and lipid metabolism of liver in largemouth bass fed high starch (mean ± SD, *n* = 9).

Items	Days of high starch diet							
	D0	D1	D3	D5	D7	AN	LT	QT
GLUT2 ^A^	3.84 ± 0.38 ^c^	4.26 ± 0.17 ^bc^	4.86 ± 0.20 ^b^	7.88 ± 0.62 ^a^	7.60 ± 0.15 ^a^	0.000	0.000	0.000
GCK ^A^	0.26 ± 0.03 ^d^	0.44 ± 0.02 ^c^	0.53 ± 0.02 ^b^	0.73 ± 0.04 ^a^	0.53 ± 0.06 ^b^	0.000	0.003	0.000
PFK ^B^	0.47 ± 0.02 ^e^	0.89 ± 0.07 ^d^	1.48 ± 0.08 ^c^	2.11 ± 0.11 ^b^	2.33 ± 0.10 ^a^	0.000	0.000	0.000
PK ^C^	0.08 ± 0.02 ^d^	0.09 ± 0.01 ^cd^	0.11 ± 0.01 ^c^	0.16 ± 0.01 ^b^	0.2 ± 0.02 ^a^	0.000	0.000	0.000
LDH ^C^	0.51 ± 0.01 ^c^	0.78 ± 0.03 ^b^	0.78 ± 0.08 ^b^	0.81 ± 0.01 ^b^	0.98 ± 0.01 ^a^	0.000	0.156	0.001
PEPC ^D^	4.14 ± 0.09 ^e^	6.84 ± 0.14 ^d^	8.38 ± 0.66 ^c^	9.9 ± 0.42 ^b^	14.33 ± 0.37 ^a^	0.000	0.000	0.000
FBP ^E^	27.42 ± 0.79 ^c^	27.94 ± 0.84 ^c^	33.02 ± 1.00 ^b^	35.98 ± 0.87 ^a^	32.00 ± 0.84 ^b^	0.000	0.002	0.000
G6Pase ^A^	7.11 ± 0.18 ^d^	10.13 ± 0.33 ^c^	14.46 ± 0.66 ^a^	14.05 ± 0.58 ^a^	11.34 ± 0.27 ^b^	0.000	0.002	0.001
GCS ^F^	31.41 ± 0.99 ^c^	41.20 ± 0.86 ^b^	41.16 ± 1.59 ^b^	50.32 ± 1.08 ^a^	33.32 ± 1.56 ^c^	0.000	0.066	0.085
GBE ^G^	16.49 ± 0.18 ^c^	16.30 ± 0.32 ^c^	25.45 ± 0.90 ^a^	17.81 ± 0.67 ^b^	17.06 ± 0.43 ^bc^	0.000	0.082	0.019
GDE ^G^	32.51 ± 1.28 ^a^	23.84 ± 1.28 ^b^	23.14 ± 1.05 ^b^	21.12 ± 1.34 ^bc^	19.16 ± 1.46 ^cd^	0.000	0.024	0.000
LAG ^H^	48.25 ± 0.60 ^e^	85.80 ± 0.11 ^d^	102.08 ± 0.90 ^c^	104.97 ± 0.59 ^b^	109.49 ± 0.13 ^a^	0.000	0.000	0.000
LA ^I^	0.52 ± 0.01 ^d^	0.77 ± 0.02 ^c^	0.97 ± 0.01 ^a^	0.9 ± 0.01 ^b^	0.75 ± 0.01 ^c^	0.000	0.085	0.000
PA ^J^	0.1 ± 0.01 ^d^	0.19 ± 0.01 ^c^	0.36 ± 0.01 ^a^	0.37 ± 0.01 ^a^	0.3 ± 0.01 ^b^	0.000	0.002	0.000
Ac-CoA ^A^	4.50 ± 0.38 ^c^	4.15 ± 1.21 ^c^	5.04 ± 0.08 ^c^	9.56 ± 0.83 ^b^	10.90 ± 0.19 ^a^	0.000	0.475	0.000
ACC ^K^	1.27 ± 0.04 ^d^	1.27 ± 0.03 ^d^	1.44 ± 0.02 ^c^	1.53 ± 0.03 ^b^	2.01 ± 0.03 ^a^	0.000	0.000	0.000
FAS ^L^	48.00 ± 0.71 ^e^	81.69 ± 0.36 ^d^	102.68 ± 0.79 ^a^	93.87 ± 0.56 ^c^	98.08 ± 0.73 ^b^	0.000	0.000	0.000
Mal-CoA ^J^	287.40 ± 24.89 ^d^	335.78 ± 11.55 ^c^	366.26 ± 13.63 ^bc^	443.40 ± 15.15 ^a^	392.92 ± 25.33 ^ab^	0.000	0.000	0.034
LTAG ^I^	0.13 ± 0.02 ^c^	0.13 ± 0.01 ^c^	0.21 ± 0.01 ^b^	0.25 ± 0.01 ^a^	0.23 ± 0.01 ^ab^	0.000	0.001	0.000

Note: ^a–e^ Values in the same row with different letters indicate significant differences (*p* < 0.05). A = ng/mg prot, B = U/mg, C = U/g prot, D = IU/L, E = U/mg prot, F = nmol/min/mg prot, G = IU/g prot, H = mg/g, I = mmol/g prot, J = μmol/mg prot, K = mmol/h/mg prot, L = U/L.

**Table 6:** Effects of dietary days on the antioxidative and oxidative indices of liver in largemouth bass fed high starch (mean ± SD, *n* = 9).

Items	Days of High Starch Diet							
	D0	D1	D3	D5	D7	AN	LT	QT
T-SOD ^A^	17.34 ± 0.50 ^a^	12.81 ± 0.80 ^b^	12.79 ± 0.54 ^b^	12.94 ± 0.51 ^b^	10.72 ± 0.61 ^c^	0.000	0.228	0.065
CAT ^A^	18.62 ± 1.00 ^a^	15.13 ± 0.62 ^b^	13.27 ± 0.94 ^bc^	11.78 ± 1.47 ^c^	12.01 ± 0.57 ^c^	0.000	0.004	0.000
GST ^A^	45.24 ± 1.78 ^c^	42.79 ± 1.15 ^c^	47.71 ± 1.90 ^c^	61.04 ± 2.67 ^b^	73.63 ± 2.68 ^a^	0.000	0.000	0.000
ROS ^B^	5.08 ± 0.13 ^a^	6.57 ± 0.14 ^b^	7.69 ± 0.13 ^c^	8.26 ± 0.15 ^d^	7.74 ± 0.15 ^c^	0.000	0.000	0.000
H_2_O_2_^C^	16.11 ± 0.12 ^e^	20.01 ± 0.21 ^d^	22.59 ± 0.31 ^c^	34.04 ± 0.47 ^a^	27.74 ± 0.65 ^b^	0.000	0.000	0.022
T-AOC ^C^	0.56 ± 0.06 ^a^	0.47 ± 0.09 ^ab^	0.44 ± 0.07 ^ab^	0.36 ± 0.08 ^bc^	0.26 ± 0.07 ^c^	0.000	0.014	0.000
MDA ^D^	9.52 ± 0.49 ^c^	12.81 ± 0.80 ^b^	12.79 ± 0.54 ^b^	12.94 ± 0.51 ^b^	17.34 ± 0.50 ^a^	0.002	0.012	0.032

Note: ^a–e^ Values in the same row with different letters indicate significant differences (*p* < 0.05). A = U/mg prot, B = mg/mL, C = mmol/g prot, D = nmol/mg prot.

**Table 7:** Effects of dietary days on the anti-inflammatory and pro-inflammatory cytokines of liver in largemouth bass fed high starch (mean ± SD, *n* = 9).

Items	Days of High Starch Diet							
	D0	D1	D3	D5	D7	AN	LT	QT
TNF-α ^A^	160.77 ± 11.54 ^e^	280.00 ± 7.69 ^d^	644.10 ± 8.01 ^c^	704.36 ± 18.18 ^b^	749.23 ± 10.18 ^a^	0.000	0.000	0.000
IL-6 ^A^	86.49 ± 3.94 ^d^	155.24 ± 3.94 ^c^	239.27 ± 3.61 ^a^	190.31 ± 5.80 ^b^	192.40 ± 9.26 ^b^	0.000	0.013	0.000
IL-8 ^B^	77.13 ± 1.21 ^c^	106.21 ± 2.26 ^b^	124.71 ± 1.39 ^a^	126.67 ± 4.28 ^a^	106.32 ± 3.92 ^b^	0.000	0.034	0.000
IL-12 ^A^	106.48 ± 1.44 ^b^	159.62 ± 1.63 ^a^	165.28 ± 9.98 ^a^	164.34 ± 6.19 ^a^	166.86 ± 4.84 ^a^	0.000	0.006	0.000
IFN-γ ^A^	67.16 ± 4.31 ^b^	65.93 ± 11.77 ^b^	69.12 ± 13.07 ^b^	106.65 ± 3.77 ^a^	115.44 ± 4.41 ^a^	0.000	0.000	0.000
IL-17 ^A^	6.20 ± 0.85 ^e^	19.83 ± 0.26 ^d^	43.21 ± 1.29 ^b^	51.35 ± 1.06 ^a^	29.59 ± 0.95 ^c^	0.000	0.010	0.000
IL-1β ^A^	25.06 ± 3.27 ^d^	36.00 ± 1.78 ^c^	52.88 ± 2.42 ^a^	54.48 ± 2.85 ^a^	47.30 ± 1.15 ^b^	0.000	0.001	0.000

Note: ^a–e^ Values in the same row with different letters indicate significant differences (*p* < 0.05). A = ng/L, B = pg/L.

## Data Availability

The data presented in this study are available in the main article.
